# March of Dimes Foundation: leading the way to birth defects prevention

**DOI:** 10.1186/s40985-017-0058-3

**Published:** 2017-05-12

**Authors:** Salimah R. Walani, Janis Biermann

**Affiliations:** 1 Global Health Programs, March of Dimes Foundation, White Plains, NY USA; 2 Education & Health Promotion, March of Dimes Foundation, White Plains, NY USA

**Keywords:** March of Dimes, Birth defects, Preterm birth, Babies, Zika, Prematurity

## Abstract

Birth defects are a major cause of mortality among children under five. In accordance with its mission, the March of Dimes Foundation is dedicated to reducing the toll of birth defects on children, families, and society. Founded in 1938 to fight polio, March of Dimes currently focuses on prevention of birth defects and preterm birth and has had a major influence on surveillance, research, advocacy, awareness, and education related to birth defects prevention and care. In the USA, it has played an active role in promoting and advocating for newborn screening for early diagnosis and treatment of congenital disorders, folic acid fortification of grains for prevention of neural tube defects, and more recently on raising awareness about birth defects related to Zika virus infection. March of Dimes has played a major role in promoting prevention of birth defects globally by publishing data-based reports and papers related to the toll of birth defects and by supporting surveillance and preconception health education programs. March of Dimes birth defects health education materials directed for raising awareness among families are used worldwide. Additionally, March of Dimes had equipped health care workers and policy makers with essential information about birth defects through published materials and sponsoring of conferences that allow for networking and knowledge exchange. March of Dimes remains committed to prevention of birth defects through supporting research related to causes of birth defects, empowering women and girls with health knowledge, and advocating for policies and programs at national and global levels for giving every child an opportunity to attain his or her optimal level of health.

## Background

Close to eight million babies worldwide are born with a serious birth defect every year and more than three million of them die before age five [1]. Under its current mission to improve the health of babies by preventing birth defects, premature birth, and infant mortality, the March of Dimes Foundation is the leading nonprofit organization dedicated to reducing the toll taken by birth defects on children, families, and society in the USA and globally.

The March of Dimes was established by President Franklin D. Roosevelt in 1938 to combat epidemic polio. Within the first 20 years of its existence, the March of Dimes funded the successful development of both the Salk and Sabin vaccines against this dreaded disease, leading the way to eliminating polio from most of the world.

With its longstanding history of influencing major developments to improve public health outcomes, the March of Dimes has committed in recent decades to improving maternal and infant health in the USA and globally by supporting public and professional education, advocacy, community services, and research. In 2016 alone, the March of Dimes provided more than $5 million in funding for investigator-initiated grants for cutting-edge research to identify the leading causes of birth defects and ways to improve prevention and care.

Many March of Dimes-funded initiatives for improving the health of mothers and babies in the USA have benefitted populations around the world. For example, during the 1960s, March of Dimes medical director Dr. Virginia Apgar developed the APGAR score, a system to help health professionals evaluate the physical condition of babies at birth. Today, the APGAR score is used worldwide and serves as a universally understood initial assessment tool to determine the health of a newborn. Dr. Apgar also popularized the use of the term “birth defects,” which remains widely used worldwide to describe congenital anomalies. Like Dr. Apgar, numerous other March of Dimes grantees over the years have paved the way to advances in research and care for affected children. Through determining the underlying causes of certain birth defects and identifying the risk factors associated with them, the March of Dimes promotes strategies for birth defects prevention.

The March of Dimes believes that every baby should receive the best care possible to help them achieve their best health. It is this belief that led us to become a prominent voice in advocacy for the creation and expansion of uniform newborn screening programs [2] to detect metabolic disorders and other conditions not visible at birth that may cause catastrophic health problems or death if not diagnosed and treated immediately. Although early diagnosis and care of affected children are essential, the March of Dimes remains a strong proponent of efforts for prevention of birth defects. For example, in the early 1990s, in response to the emerging evidence that consuming folic acid before pregnancy and into the first trimester could greatly reduce a woman’s risk of having a baby with a neural tube defect (NTD), the March of Dimes led efforts to persuade the US Food and Drug Administration to require folic acid fortification of the grain food supply. As a result of the mandatory folic acid fortification in the USA, NTDs in the USA have declined by about one third since fortification began in 1998 [3].

Two landmark reports published by the March of Dimes and partners have shown that the burden of birth defects [1] and premature birth [4] is specifically high in low- and middle-income countries of the world. Similarly, studies [5] have shown that babies in the USA have different chances of surviving and thriving simply based on the circumstances of their birth, i.e., their families’ socio-economic status, race, and ethnicity, as well as their geographic location. The March of Dimes is leading a new movement for equity by promoting health for everyone, regardless of their income, education, racial/ethnic background, or health insurance status. Our goal is ensure that every baby gets a fair chance for a healthy start in life.

## Initiatives related to birth defects

### Highlights of the US-based birth defects efforts

When the March of Dimes initiated its mission to prevent birth defects, few people realized the scope or complexity of the problem. However, knowledge rapidly expanded through conferences sponsored by the March of Dimes to foster exchanges among researchers and scientists from diverse disciplines. One such conference, which continued on an annual basis, is the Human and Mammalian Genetics and Genomics Course at Jackson Laboratories in Bar Harbor, Maine, now in its 58th year. The March of Dimes provided support for research and medical services in previously unexplored areas, such as the study of human chromosomes.

In 1953, the March of Dimes gave a grant to James D. Watson who, with Francis Crick, later identified the double helix structure of DNA. The pair won the Nobel Prize in 1962 and paved the way for modern genetic medicine, including the mapping of the human genome. The March of Dimes also established a network of birth defects centers in university and teaching hospitals around the country to provide, for the first time, early diagnosis and comprehensive care for babies born with birth defects.

In the early 1960s, March of Dimes grant funding enabled Dr. Robert Guthrie, who had developed the first newborn blood test for PKU (phenylketonuria), to produce a mass screening test for the disorder. The March of Dimes and patient/family groups went on to campaign tirelessly for expanded and standardized state newborn screening programs nationwide. Today, every baby born in every state in the USA receives screening for a uniform panel of conditions that can be treated soon after birth to prevent intellectual disability or death.

In 1973, the March of Dimes published the first *Birth Defects Atlas and Compendium,* which standardized the names and descriptions of 842 birth defects. Also in the 1970s, the March of Dimes initiated a regional system of neonatal intensive care units (NICUs) in which babies with birth defects or born prematurely could receive the best care.

Other notable accomplishments by researchers funded by the March of Dimes included the first report showing that drinking alcohol during pregnancy could cause fetal alcohol syndrome and the development of surfactant therapy for babies born with immature lungs that has saved the lives of hundreds of thousands.

Also in the early 1970s, the March of Dimes launched a new office of public affairs in Washington, DC, to coordinate advocacy for state and US federal legislation to further prevention, education, and care for babies with birth defects. The March of Dimes has had many advocacy successes over the years, including the passage of the State Children’s Health Insurance Program (SCHIP), ensuring health care coverage for up to five million children; the Birth Defect Prevention Act, establishing a National Center on Birth Defects and Developmental Disabilities; and the Newborn Screening Saves Lives Act. The most recent advocacy achievement was winning the Food and Drug Administration’s agreement to voluntary fortification of corn masa flour with folic acid to further help reduce the incidence of NTD.

While pursuing a robust national policy agenda for birth defects prevention and care, the March of Dimes sought to raise public consciousness on birth defects and teach known ways of prevention. The organization placed public service announcements in popular magazines and published dozens of informational materials. In the early 1980s, the March of Dimes launched its first program for advancing the overall health of women and babies. *Babies & You* was a prenatal education program delivered in the workplace, with classes taught by March of Dimes staff or volunteers. This program has evolved into other educational offerings for women in the workplace available today.

 In 1992, US Public Health Service recommended that all women of childbearing age capable of becoming pregnant consume 400 micrograms of folic acid daily to reduce their risk for having a baby affected by an NTD. Following that, the March of Dimes launched its first national campaign urging women to take a multivitamin containing folic acid every day. This important preconception health message evolved into an enhanced Folic Acid Campaign launched in 1999 in collaboration with the Centers for Disease Control and Prevention (CDC), the American College of Obstetricians, the American Academy of Pediatrics, and the Spina Bifida Association of America. Providing education for women, health care providers and the public to urge compliance with folic acid consumption guidelines for reducing risk of NTDs was the main focus of the campaign, which ran from 1999 to 2002. This campaign had a major impact in increasing awareness of the importance of preconception folic acid consumption: In 1995, only 4% of women reported knowing that folic acid can prevent birth defects, and in 2004, 24% of women reported the same [6]. Building on this momentum, the March of Dimes and its partner organizations advocated for folic acid fortification of enriched grain products. Mandatory fortification of wheat flour became a law in 1996, taking effect in 1998, and resulting in a 35% decrease [3] in the rate of NTDs in the USA from 1999 to 2011 [7].

After tremendous successes with campaigns for birth defects prevention, the March of Dimes decided to launch a new campaign to decrease the rate of preterm birth in the USA in 2003. This Prematurity Campaign supports research, education, community service, and advocacy initiatives—the same strategies that yielded so many discoveries and advances for birth defects prevention. One of the signature programs of this Prematurity Campaign is the March of Dimes NICU Family Support Program®, which provides comfort and information to families with a baby in the NICU, whether born preterm or with a birth defect. More than 75,000 families in the USA each year are helped by this program.

The March of Dimes commitment to birth defects prevention is such that we are always responsive to emerging challenges. For example, after the first article appeared in *The New York Times* on December 28, 2015, [8] calling attention to the Zika virus and its threat to pregnancy, the March of Dimes decided it was time to inform women and men of childbearing age and the general public about a possible Zika virus epidemic. Educational information was developed for consumers and made available widely on our websites at www.marchofdimes.org/zika and in Spanish on www.nacersano.org/zika. Health information specialists at the March of Dimes answered email queries about Zika infection and promoted prevention messages via social media. The March of Dimes held various media events to raise journalists’ awareness about Zika and its potential impact on babies, and the March of Dimes became a leader in the fight against the spread of the virus. After months of intense advocacy by the March of Dimes and the coalition of more than 90 organizations that we headed, Congress finally passed a $1.1 billion Zika funding bill in September 2016. Working closely with the CDC and many other partners, the March of Dimes continues to promote Zika virus prevention while helping to develop information about services for families affected by Zika.

### Salient global efforts related to birth defects

In 1998, the March of Dimes decided to extend its mission globally through formation of a Global Programs Department. Although the March of Dimes does not have any international offices or staff in any other countries outside the USA, it is well-known and well-respected worldwide for its contributions to prevention of birth defects and premature birth. The March of Dimes conducts its global work mainly through partnerships with international and in-country organizations that work in Reproductive, Maternal, Newborn, Child and Adolescent Health (RMNCAH). As a “Non-state Actor in Official Relations” with the World Health Organization (WHO) and with Pan American Health Organization (PAHO), the March of Dimes has engaged in high-level scientific, technical, and policy discussions related to improving the health of mothers and babies globally. Additionally, we have supported and engaged local academic institutions in low-and middle-income countries to advance the health of women and girls through preconception health activities [9].

One of the major contributions of the March of Dimes globally was to bring to light the high worldwide toll of birth defects through the publication in 2006 of the first-ever country-level estimates of serious birth defects of genetic origin with data from 193 countries [1]. This report has served to advance engagement of researchers, practitioners, and policy makers in addressing the widely neglected issue of birth defects. The report is further believed to be the impetus for the World Health Organization’s Birth Defects Resolution in 2010 that urged United Nations member states to engage in birth defects awareness, surveillance, prevention, and care-related activities through development of national plans [10].

Recognizing the need to build capacity in lower-income countries for prevention and care related to birth defects, in 2001, March of Dimes began sponsoring a biennial International Conference on Birth Defects and Disabilities in the Developing World. To date, these conferences have been held in Tanzania (2015), the Philippines (2013), Poland (2011), India (2009), Brazil (2007), China (2005), and South Africa (2001). These conferences are usually attended by 300–400 participants and serve as a platform for discussions, knowledge sharing, and action. For example, the 2015 conference in Tanzania led to the publication of a consensus statement on “Prevention of Congenital Disorders and Care of Affected Children” [11] signed by over 75 conference participants. The 8th conference in this series is being planned to be held in Bogota, Colombia in November 2017 and will address the ongoing epidemic of Zika virus-related birth defects, among other topics.

The March of Dimes is a world-renowned leader in raising birth defects awareness. On March 3, 2015, March of Dimes joined 11 other organizations to launch the first-ever World Birth Defects Day, providing a platform for governmental and non-governmental agencies to raise awareness about birth defects globally. In 2017, the third annual World Birth Defects Day efforts were formally joined by close to 80 organizations from about 35 countries that officially participated in raising awareness about surveillance, prevention, and care related to birth defects. This effort resulted in over 24 million impressions on Twitter and other key social media indicators related to the event.

## Conclusion

The March of Dimes has had a critical role in raising awareness of birth defects in the USA and globally, and has played a prominent role in influencing policies and programs for prevention of birth defects around the world. From initially coining the term “birth defects” to advocating for mandatory folic acid fortification for prevention of NTDs, to promoting education for prevention of Zika virus-related birth defects, the March of Dimes has demonstrated its leadership in the field of birth defects.

The March of Dimes is focusing today on empowering women of childbearing age with health education. We believe that our mission to prevent birth defects and premature birth can be fully realized only by enabling women to improve their own health and that of their families. This has led us to develop health education information in plain language for women of childbearing age in both English and Spanish. We reach women globally through a variety of media, including print, web, video, and social media. We have trained health information specialists who answer questions and provide information through a variety of online channels. In low- and middle-income countries, we support programs for improving preconception health knowledge of women and girls in schools, workplaces, and communities. And in addition to consumer education, we have created tools and resources for health care professionals, healthcare organizations, and policymakers that are used worldwide.

Throughout its nearly 80 years of existence, the March of Dimes has maintained three core values: bringing science into service for people, building the public trust, and harnessing the power of volunteers. These core values continue to guide March of Dimes staff, volunteers, and corporate sponsors to support community service programs, advocacy, research, and education on birth defects prevention and care. Until we find ways to solve these conditions, we will not cease our efforts to improve the quality of life for all those who are affected by them.

## References

[1] Christianson A, Howson C, Modell B. March of Dimes global report on birth defects: the hidden toll of dying and disabled children. The March of Dimes Birth Defects Foundation. 2006. http://www.marchofdimes.org/materials/global-report-on-birth-defects-the-hidden-toll-of-dying-and-disabled-children-executive-summary.pdf. Accessed 9 March 2017.

[2] Howse JL, Weiss M, Green NS. Critical role of the March of Dimes in the expansion of newborn screening. PubMed. 2006. PMID: 17183577; doi: 10.1002/mrdd.20129. https://www.ncbi.nlm.nih.gov/pubmed/17183577. Accessed 9 Mar 2017. 10.1002/mrdd.20129 17183577

[3] Folic Acid: Birth Defects COUNT. Neural tube defects. Centers for Disease Control and Prevention. 2016. https://www.cdc.gov/ncbddd/birthdefectscount/basics.html. Accessed 9 Mar 2017.

[4] Howson CP, Kinney MV, Lawn JE. Born too soon: the global action report on preterm birth. The March of Dimes Foundation, PMNCH, Save the Children, World Health Organization. 2012. http://www.who.int/pmnch/media/news/2012/preterm_birth_report/en/. Accessed 9 Mar 2017.

[5] Vanderbilt AA, Wright MS (2013). Infant mortality: a call to action overcoming health disparities in the United States. Med Educ Online.

[6] Folic acid and the prevention of birth defects. National Survey of Pre-Pregnancy Awareness and Behavior Among Women of Childbearing Age 1995-2004. The Gallup Organization for March of Dimes Birth Defects Foundation. 2004. 31-1897-04. p. 3.

[7] Williams J, Mai CT, Mulinare J, Isenburg J, Flood TJ, Ethen M, Frohnert B, Russell KS (2015). Updated estimates of neural tube defects prevented by mandatory folic acid fortification—United States, 1995–2011. Centers for Disease Control and Prevention. MMWR.

[8] McNeil DG Jr. Zika virus, a mosquito-borne infection, May Threaten Brazil’s. The New York Times. December 28, 2015. https://www.nytimes.com/2015/12/29/health/zika-virus-brazil-mosquito-brain-damage.html. Accessed 9 Mar 2017.

[9] Charafeddine L, El Rafei R, Azizi S, Sinno D, Alamiddine K, Howson CP, Walani RS, Ammar W, Nassar A, Yunis K (2014). Improving awareness of preconception health among adolescents: experience of a school-based intervention in Lebanon. BMC Public Health.

[10] World Health Organization. Birth defects. 2010. http://apps.who.int/gb/ebwha/pdf_files/WHA63/A63_R17-en.pdf. Accessed 9 Mar 2017.

[11] Darmstadt GL, Howson CP, Walraven G, Armstrong RW, Blencowe HK, Christianson AL, Kent A, Malherbe H, Murray JC, Padilla CD, Walani SR (2016). Prevention of congenital disorders and care of affected children: a consensus statement. JAMA Pediatr.

